# Evaluation of the P-Δ (P-Delta) effect in columns and frames using the two-cycle method based on the solution of the beam-column differential equation

**DOI:** 10.1016/j.mex.2023.102248

**Published:** 2023-06-09

**Authors:** Rodrigo Bird Burgos, Lucas Encarnação Silva

**Affiliations:** a Department of Structures and Foundations, Rio de Janeiro State University, R. São Francisco Xavier, 524, Maracanã, Rio de Janeiro, Brazil; b Civil Engineering Post Graduate Program, Rio de Janeiro State University, R. São Francisco Xavier, Maracanã 524, Rio de Janeiro, Brazil

**Keywords:** Second order analysis, Geometric nonlinearity, Slender bars, Buckling, Structural instability, Two-cycle with “exact” interpolation functions for geometrically nonlinear analysis

## Abstract

P-Delta is a nonlinear phenomenon that results from the consideration of axial loads acting on the deformed configuration of a member of the structure, usually a beam-column. This effect is especially significant in slender members, which can undergo large transversal displacements which tend to increase the bending moment caused by an axial load P acting upon them. The P-delta effect can be computed through a geometrically nonlinear analysis, usually employing the Finite Element Method, which subdivides each bar of the frame in finite segments known as elements. Since discretization (subdivision) and the use of iterative schemes (like Newton-Raphson) are sometimes undesirable, especially for students, avoiding it can be didactically interesting. This work proposes the use of a new approach to perform a simplified nonlinear analysis using the two-cycle method and a tangent stiffness matrix obtained directly from the homogeneous solution of the problem's (beam-column) differential equation. The proposed approach is compared to the results obtained by the traditional two-cycle method which uses geometric and elastic stiffness matrices based on cubic (Hermitian) polynomials and a P-Delta approximation using the pseudo (fictitious) lateral load method.

## Review Highlights

• Tangent stiffness matrix based on the analytical solution of the problem's differential equation; all parameters of the model are present in only one set of coefficients.
• No need for discretization of more than one element per bar; a reliable nonlinear analysis is performed with only two steps, without the use of incremental schemes like load or displacement control.
• Results for single members are identical to the analytical exact solutions of the differential equation.

## Graphical abstract

**Figure:**
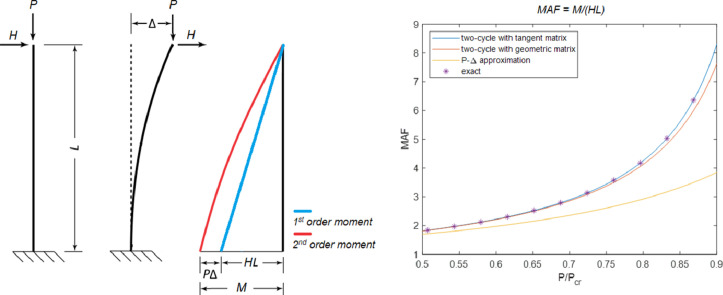

Specifications table

**Table:** 

Subject area:	Engineering
More specific subject area:	Structural analysis (Civil Engineering)
Name of the reviewed methodology:	Twocycle method with tangent stiffness matrix based on DE solutions
Keywords:	Second order analysis, geometric nonlinearity, slender bars, buckling, structural instability
Method name:	Two-cycle with “exact” interpolation functions for geometrically nonlinear analysis
Resource availability:	N/A
Review question:	N/A

## Introduction

The P-delta is a second-order (nonlinear) effect that occurs when an axial load acts upon a lateral displacement generating an additional moment and is considered a geometrically induced nonlinearity. A complete geometric nonlinear analysis requires the use of a numerical scheme, like the Finite Element Method (FEM), which involves discretization of the structural members [1]. Discretization is one of the key aspects in FEM analysis, and it is needed when interpolation functions within the elements are not the exact solution of the problem's differential equation [2]. In traditional frame elements, interpolation functions are cubic polynomials, and the exact solution is represented by trigonometric functions.

When equilibrium of forces and moments is imposed in an undeformed infinitesimal beam element the following differential equation is obtained:

(1) $$\begin{array}{ccc} & & EI\frac{d^{4}v}{dx^{4}}=0\\ & & v\left(x\right)=Ax^{3}+Bx^{2}+Cx+D. \end{array}$$

Equation (1) is obtained assuming that any bending moment arising from the axial load in the element acting upon its displacements is not large enough as to alter its behavior. Cubic polynomials are the solution of the beam problem in this case. In this context, v(x) is the curve that describes the transversal displacement of the beam at position x, E is the modulus of elasticity (Young's modulus), and I is the moment of inertia of the cross-section.

Geometric nonlinearity is especially significant in slender members, which can undergo large transversal displacements which tend to change the way loads act upon the structure [3]. The change in the bending moment caused by an axial load acting upon the deformed configuration of the structure is known as the P-delta effect and its consideration is crucial when the axial load is compressive. The differential equation that models this kind of behavior and its solution, for a compressive axial load P, are:

(2) $$\begin{array}{ccc} & & EI\frac{d^{4}v}{dx^{4}}-P\frac{d^{2}v}{dx^{2}}=0\\ & & v\left(x\right)=A\sin\left(\mu x\right)+B\cos\left(\mu x\right)+Cx+D,\;\mu =\sqrt{\frac{P}{EI}}. \end{array}$$

A detailed discussion on how to obtain these equations and solutions is given in [4]. Since trigonometric functions are difficult to deal with in numerical schemes due to the possibility of singularity in the matrices involved, the usual way to capture this behavior is to subdivide the domain into finite elements obtained using the cubic solutions of Eq. (1) and performing an incremental analysis to take the nonlinearity introduced by the axial load into account. Since discretization and the use of iterative schemes (like Newton-Raphson) are sometimes undesirable, especially for students, avoiding it can be didactically interesting. This work proposes the use of a different stiffness matrix in a two-cycle FEM analysis to represent displacements and bending moments. The matrix coefficients are obtained from the homogeneous solution of the differential equation of an axially loaded beam-column, following the work of Burgos and Martha [5]. Examples for single columns and a two-bar frame were analyzed and comparisons were made using reference analytical and numerical solutions.

## P-Delta effect

**Fig. 1:**
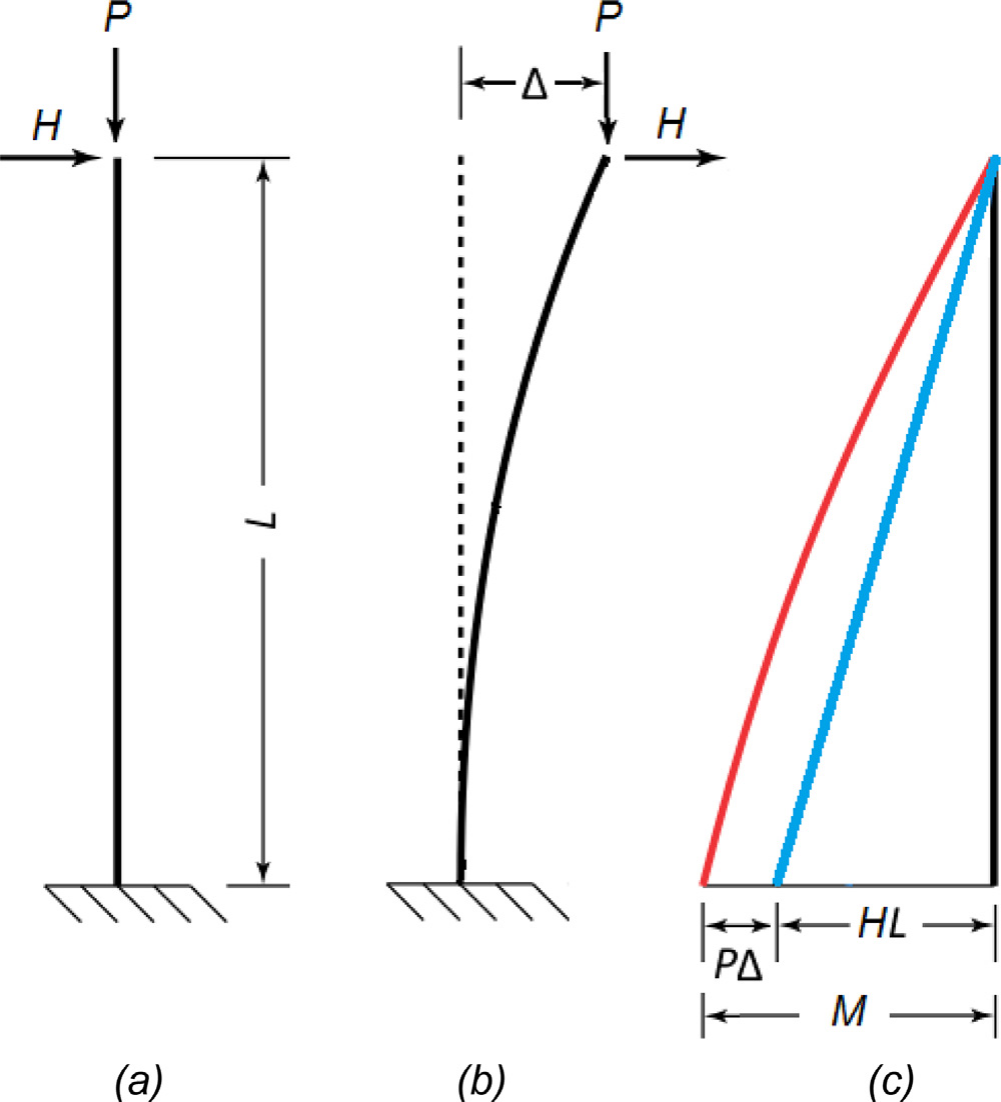
P-Delta effect (adapted from [8]): (a) undeformed configuration; (b) deformed configuration: (c) bending moment distribution.

A typical P-Delta representation is shown in Fig. 1. Linear structural analysis separates axial and transversal effects since it considers that displacements are small as not to contribute to a significant change in the bending moment. It this case, the undeformed configuration (Fig. 1-a) is used as the reference, the bending moment is given by HL and is known as the first order moment (blue curve in Fig. 1-c). If the displacement Δ or the axial load P (or both) are large enough, the P-Δ component of the bending moment M cannot be neglected (red curve in Fig. 1-c). In this case, the total bending moment M is known as the second order moment and is given by the sum of the first order and the P-Δ components. The reference configuration is the deformed one (Fig. 1-b). Since the displacement Δ depends on the transversal load H, the second order moment M can be written in terms of the first order moment HL and the ratio between them is called MAF (Moment Amplification Factor) (Eq. (3)). It is usual to find references to the MAF instead of the bending moment itself in the literature [6,7].

(3) M‾secondordermoment=MAF×HL‾firstordermoment

## Approximate P-Delta analysis (Pseudo Load) and Two-cycle approach

One of the easiest and most used methods for evaluating the P-Delta effect is que Pseudo Lateral Load Method [9]. It assumes that the axial load P acting upon the displacement Δ obtained from a linear analysis can be used as a first approximation of the additional bending moment. Then, a fictitious lateral load that would cause the same moment is applied, hence giving a new linear displacement and the process is repeated iteratively until a tolerance is reached. Since this process can be seen as a geometric progression, with ratio given by the relation between the additional moment (P-Δ) and the first order moment (M_1_), the result of the iterative method is the sum of this progression [10]. Eq. (4) shows the Moment Amplification Factor (MAF) formula when the Pseudo Load Method is used.

(4) $${\mathrm{MAF}}_{P-\Delta }=\frac{1}{1-\frac{\Delta M_{P-\Delta }}{M_{1}}}$$

When the case shown in Fig. 1 is considered, the displacement Δ is given by HL^3^/3EI and the additional moment is then given by PHL^3^/3EI. Since the first order moment is given by M_1_ = HL, the resultant MAF is shown in Eq. (5).

(5) $${\mathrm{MAF}}_{P-\Delta }=\frac{1}{1-\frac{P\frac{HL^{3}}{3EI}}{HL}}=\frac{1}{1-\frac{PL^{2}}{3EI}}$$

It is clear from Eq. (5) that the MAF is nonlinearly dependent on P, i.e., the consideration of the bending moment produced by the axial load generates a considerable raise in the total moment at the base of the column. It is also clear that a value of P =3EI/L^2^ takes the MAF value to infinity. It is a reasonable approximation since the critical load for this column is P_cr_ = π^2^EI/4L^2^ = 2.47EI/L^2^.

In a nonlinear elastic analysis based on the FEM, the structure can be analyzed in an incremental scheme according to [K_t_]{d∆}={dF}, where {d∆} is a vector of incremental nodal displacements and rotations, {dF} is a vector of incremental nodal forces, and [K_t_] is a tangent stiffness matrix, which is composed by a linear elastic component, [K_e_], and a nonlinear part, [K_g_], known as the geometric matrix [11]. Members of the structure need to be divided into several elements and the increment in loads and/or displacements needs to be small enough to provide satisfactory results. The conventional formulation of the components of the tangent stiffness matrix is performed using the virtual work principle using cubic (Hermitian) polynomials and disregarding higher-order terms in the strain tensor. The expressions for both the elastic and geometric stiffness matrices are given in Eq. (6).

(6) $$\begin{array}{c} K_{t}=K_{e}+K_{g}\\ K_{e}=\left[\begin{array}{cccc} \frac{12EI}{L^{3}} & \frac{6EI}{L^{2}} & -\frac{12EI}{L^{3}} & \frac{6EI}{L^{2}}\\ \frac{6EI}{L^{2}} & \frac{4EI}{L} & -\frac{6EI}{L^{2}} & \frac{2EI}{L}\\ -\frac{12EI}{L^{3}} & -\frac{6EI}{L^{2}} & \frac{12EI}{L^{3}} & -\frac{6EI}{L^{2}}\\ \frac{6EI}{L^{2}} & \frac{2EI}{L} & -\frac{6EI}{L^{2}} & \frac{4EI}{L} \end{array}\right],\;K_{g}=\left[\begin{array}{cccc} -\frac{6P}{5L} & -\frac{P}{10} & \frac{6P}{5L} & -\frac{P}{10}\\ -\frac{P}{10} & -\frac{2PL}{15} & \frac{P}{10} & \frac{PL}{30}\\ \frac{6P}{5L} & \frac{P}{10} & -\frac{6P}{5L} & \frac{P}{10}\\ -\frac{P}{10} & \frac{PL}{30} & \frac{P}{10} & -\frac{2PL}{15} \end{array}\right] \end{array}$$

A simpler way to use this approach consists in the Two-cycle Method, first introduced by Chen and Lui [12]. Instead of implementing an incremental scheme, which depends on several parameters (number of steps, step size), the two-cycle approach uses a linear analysis to obtain the axial forces (first cycle) and then applies these forces in the tangent stiffness matrix (second cycle). The main difference between the two approaches is that the incremental scheme updates the deformed configuration at each step, providing more accurate results. However, if displacements are not too large or the complete equilibrium path is not of concern, this difference is negligeable.

The two-cycle method can be summarized as:

1 – Linear analysis using elastic stiffness matrix: [K_e_]{Δ} = {F};
2 – Construction of geometric component of tangent stiffness matrix, [K_g_], based on the axial forces obtained in the previous step;
3 – Tangent stiffness matrix construction: [K_t_]=[K_e_]+[K_g_];
4 – New analysis using tangent stiffness matrix: [K_t_]{Δ} = {F}.

## Tangent stiffness matrix proposed

The presented method combines the simplicity of the two-cycle approach with the accuracy of the tangent stiffness matrix obtained directly from the solution of the differential equation shown previously. In this way nonlinearity is naturally induced by the equilibrium of the infinitesimal element in the deformed configuration considering the axial effect. Therefore, the resulting matrix coefficients depend on the axial force in the element. The complete formulation and derivation of the tangent stiffness matrix is given in [5,14]. The main difference now is that there are no separate components of the tangent stiffness matrix, i.e., it presents a closed form for its coefficients, which depend on elastic parameters and axial loads, as shown in Eq. (7).

(7) $$\begin{array}{ccc} K_{t} & = & \left[\begin{array}{cccc} k_{1} & k_{2} & -k_{1} & k_{2}\\ k_{2} & k_{3} & -k_{2} & k_{4}\\ -k_{1} & -k_{2} & k_{1} & -k_{2}\\ k_{2} & k_{4} & -k_{2} & k_{3} \end{array}\right]\\ k_{1} & = & \frac{EI\mu^{3}\text{sin}\left(\mu L\right)}{2-2\text{cos}(\mu L)-\mu L\sin(\mu L)}\\ k_{2} & = & \frac{EI\mu^{2}\left[1-\cos\left(\mu L\right)\right]}{2-2\text{cos}(\mu L)-\mu L\sin(\mu L)}\\ k_{3} & = & \frac{EI\mu \left[\text{sin}\left(\mu L\right)-\mu L\cos\left(\mu L\right)\right]}{2-2\text{cos}(\mu L)-\mu L\sin(\mu L)}\\ k_{4} & = & \frac{EI\mu \left[\mu L-\text{sin}\left(\mu L\right)\right]}{2-2\text{cos}(\mu L)-\mu L\sin(\mu L)}\\ \mu & = & \sqrt{\frac{P}{EI}} \end{array}$$

The steps involved in this method are the same as in the traditional two-cycle scheme, substituting the tangent stiffness matrix by the expression in (7). The first cycle is identical in the traditional and the proposed approach, given by a linear elastic analysis using only the elastic stiffness matrix, obtaining the axial force values for each member, which will be later used in the construction of the tangent matrix applied in the second cycle. The main difference now is that there are no approximations regarding the influence of the axial load in the coefficients of the proposed tangent matrix. It is also interesting to notice that if a Taylor series expansion (up to the third degree of P) is performed on the coefficients given in Eq. (7), the first and second components (up to degree one of P) are identical to the corresponding elastic and geometric stiffness coefficients, respectively [13].

(8) $$\begin{array}{ccc} & & k_{1}≅\frac{12EI}{L^{3}}-\frac{6P}{5L}-\frac{P^{2}L}{700EI}-\frac{P^{3}L^{3}}{63000E^{2}I^{2}}\\ & & k_{2}≅\frac{6EI}{L^{2}}-\frac{P}{10}-\frac{P^{2}L^{2}}{1400EI}-\frac{P^{3}L^{4}}{126000E^{2}I^{2}}\\ & & k_{3}≅\frac{4EI}{L}-\frac{2PL}{15}-\frac{11P^{2}L^{3}}{6300EI}-\frac{P^{3}L^{5}}{27000E^{2}I^{2}}\\ & & k_{4}≅\frac{2EI}{L}+\frac{PL}{30}+\frac{13P^{2}L^{3}}{12600EI}+\frac{11P^{3}L^{5}}{378000E^{2}I^{2}} \end{array}$$

## Validation

**Fig. 2:**
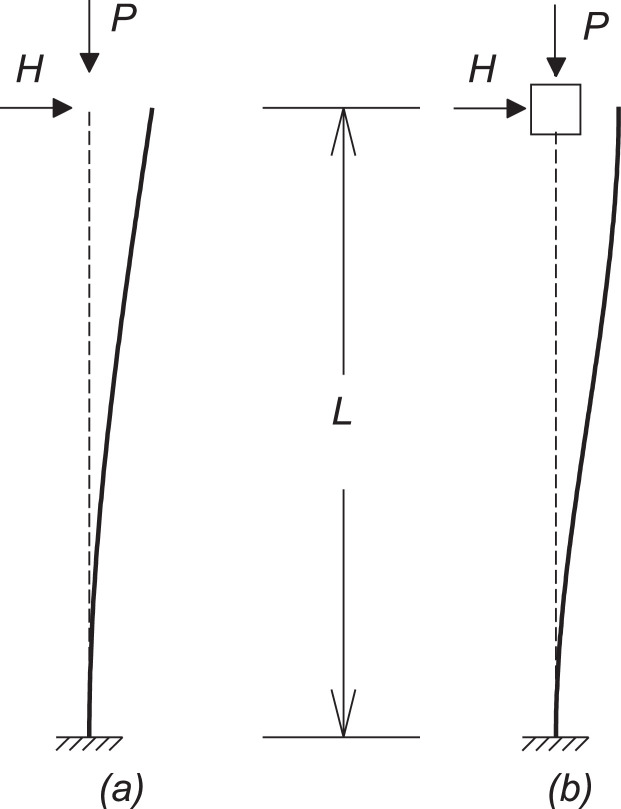
Columns used for validation of the method: (a) clamped-free; (b) clamped-fixed.

Some simple examples were assessed for the validation of the proposed method. First, columns with two different boundary conditions were modeled: clamped-free and clamped-fixed. These problems present simple analytical solutions that can be used as a reference. Fig. 2 shows both columns and their deformed configurations. In a first order (linear) analysis, the bending moment at the base is dependent solely on the lateral load H value, but when a nonlinear analysis is performed there is an amplification of this moment, which can be measured by the MAF (Moment Amplification Factor).

The clamped-free boundary condition is the classical example for the P-Delta representation, as shown in the introduction of this paper, Fig. 1. The expression for the pseudo load method was presented in Eq. (5). Its analytical solution in terms of the MAF is given by:

(9) $${\mathrm{MAF}}_{exact}=\frac{\tan\left(\mu L\right)}{\mu L},\;\mu =\sqrt{\frac{P}{EI}}.$$

The clamped-fixed boundary condition can be thought of as the superposition of two clamped-free bars. Eq. (10) shows the MAF expressions using the P-Δ approximation and the exact value.

(10) $$\begin{array}{ccc} & & {\mathrm{MAF}}_{P-\Delta }=\frac{1}{1-\frac{PL^{2}}{12EI}}\\ & & {\mathrm{MAF}}_{exact}=\frac{2-2\cos\left(\mu L\right)}{\mu L\sin\left(\mu L\right)},\;\mu =\sqrt{\frac{P}{EI}} \end{array}$$

**Fig. 3:**
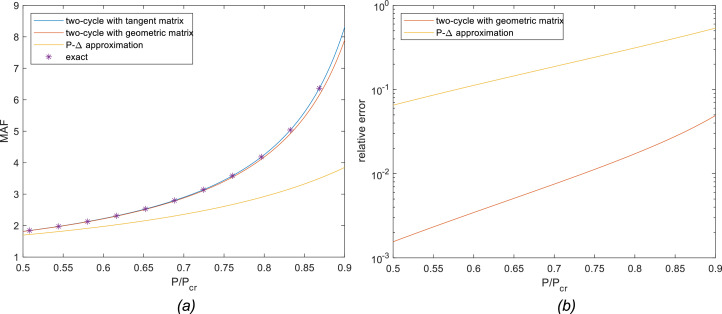
Results for the clamped-free column: (a) MAF; (b) relative error.

Fig. 3-a shows MAF vs. P/P_cr_ curves for each of the methods described in this article considering the clamped-free boundary condition. The asterisk marks are the analytical response given by Eq. (9); the blue curve represents the results obtained by the proposed method; the red one is the result obtained using geometric stiffness matrix and the yellow one was obtained from the simplified P-Δ method. For this case, the critical load P_cr_ is π^2^EI/4L^2^ ≅ 2.47EI/L^2^, as stated previously. As the axial load reaches the critical value, the approximate methods give poorer results. Fig. 3-b shows the relative error obtained for each approximate method in log scale, which is better suited for error plots. A relative error of 10^−2^ is equivalent to a 1% error since e = (MAF_ex_−MAF_num_)/MAF_ex_. The proposed method provides exact results, so the error calculation is pointless.

**Fig. 4:**
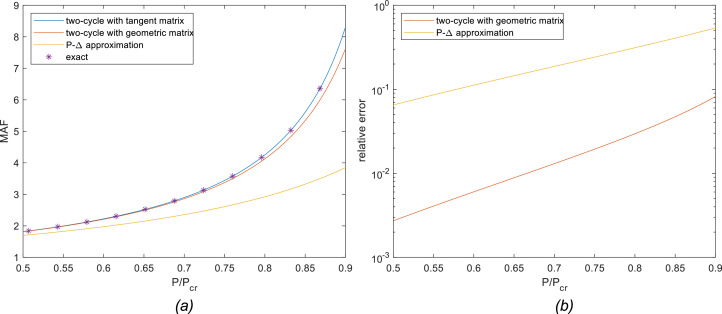
Results for the clamped-fixed column: (a) MAF; (b) relative error.

Fig. 4-a shows MAF vs. P/P_cr_ curves for each of the methods described in this article considering the clamped-fixed boundary condition. The asterisk marks are the analytical response given by Eq. (10); the blue curve represents the results obtained by the proposed method; the red one is the result obtained using geometric stiffness matrix and the yellow one was obtained from the simplified P-Δ method. For this case, the critical load P_cr_ is π^2^EI/L^2^ ≅ 9.87EI/L^2^. Fig. 4-b shows the relative error obtained for each approximate method in log scale, which is better suited for error plots. Since the degree of nonlinearity is dependent on the relation between axial load P and its critical value P_cr_, it is expected the error to increase for higher values of P/P_cr_. In the relative error plot, only geometric matrix and P-Δ approximation are shown since the proposed tangent matrix method returns the exact analytical values and error calculation is meaningless.

**Fig. 5:**
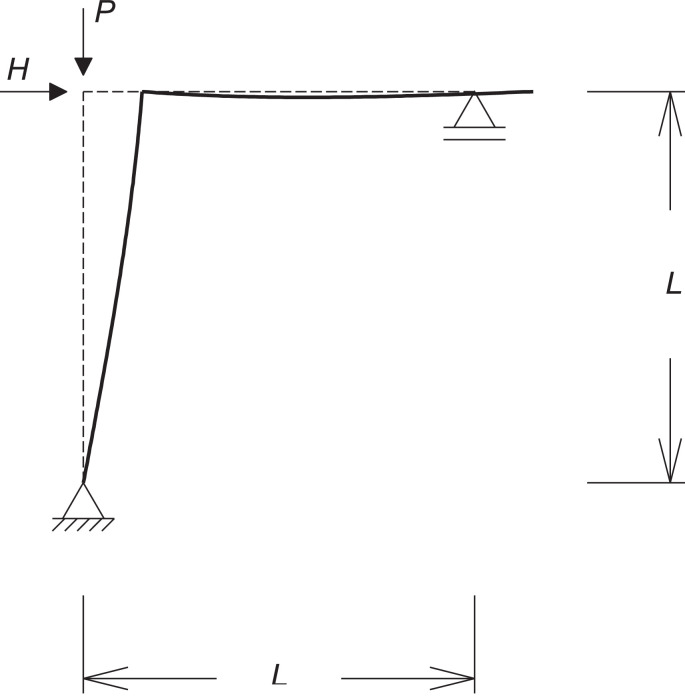
Roorda's frame.

**Fig. 6:**
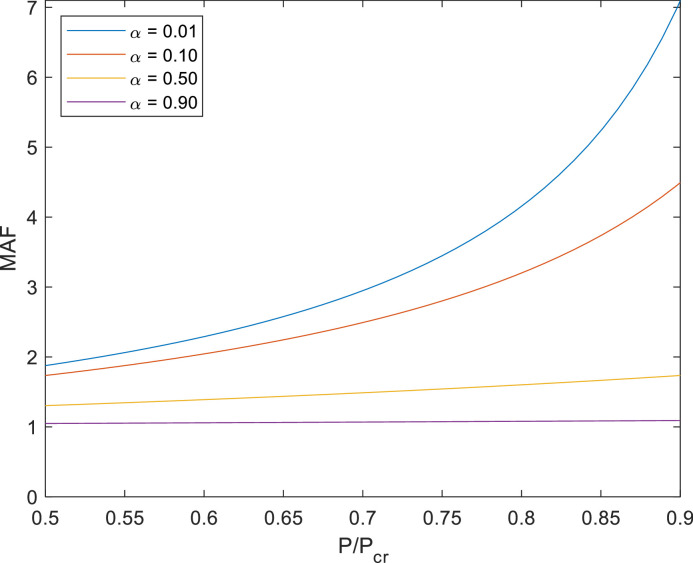
Roorda's frame results using different values of α = H/P.

As a second kind of example, a simple structure was evaluated, known as Roorda's frame, depicted in Fig. 5. For this example, only the proposed method was used since it was validated for simple columns. Differently from the columns, in this case the bars interact and the axial force in the vertical bar is dependent on the horizontal load; this will affect the coefficients of the tangent stiffness matrix. Different relations between horizontal and vertical loads (α = H/P) produce different results in terms of MAF, as shown in Fig. 6. While α can be omitted when single column expressions are obtained (MAF is independent from α), the same does not happen for frames and the relation H/P becomes crucial in determining the bending moment at the intersection between beam and column. A value of H close to P makes the axial load in the column to be almost null, which allows neglecting second-order (nonlinear) effects. This is the case of the purple curve in Fig. 6, for which H = 0.9P. The other extreme case is given by a horizontal load H so low (H = 0.01P) that the load P is fully transmitted to the column, resulting in a similar case from the two previous examples. This case is represented by the blue curve in Fig. 6. The other two cases (red and yellow curves) are obtained for intermediate α values.

In all the examples, curves for MAF values and errors were plotted from 0.5 to 0.9 because for lower values (less than 0.5) the effect of nonlinearity is negligible and for values close to unity (higher than 0.9), the bending moments tend to infinity since the critical load causes column buckling (instability).

## Final remarks

This review paper presented a different approach to obtaining second-order (nonlinear) effects in frame structures. The method proposes an alternative tangent matrix in a two-cycle approach, allowing the analyst to avoid subdivision of members and iterative schemes, which is especially useful for students and researchers interested in a simplified method to analyze the P-Delta effect. This “new” tangent stiffness matrix is obtained directly from the homogeneous solution of the problem's differential equation. For that reason, results for single columns are identical to analytical solutions. Results were excellent when compared to the traditional approach, which uses geometric stiffness matrix and requires discretization (subdivision), and a simplified P-Delta fictitious load method.

## Ethics statements

Ethical guidelines are complied with by the authors. No data was collected from social media platforms.

## CRediT authorship contribution statement

**Rodrigo Bird Burgos:** Conceptualization, Methodology, Writing – original draft, Funding acquisition. **Lucas Encarnação Silva:** Validation, Writing – review & editing.

## Declaration of Competing Interest

The authors declare that they have no known competing financial interests or personal relationships that could have appeared to influence the work reported in this paper.

## Data Availability

No data was used for the research described in the article.
